# Effect of Pentacyclic Guanidine Alkaloids from the Sponge *Monanchora pulchra* on Activity of α-Glycosidases from Marine Bacteria

**DOI:** 10.3390/md17010022

**Published:** 2019-01-01

**Authors:** Irina Bakunina, Galina Likhatskaya, Lubov Slepchenko, Larissa Balabanova, Liudmila Tekutyeva, Oksana Son, Larisa Shubina, Tatyana Makarieva

**Affiliations:** 1 G.B. Elyakov Pacific Institute of Bioorganic Chemistry, Far Eastern Branch, Russian Academy of Sciences, Vladivostok 690022, Russia; galin56@mail.ru (G.L.); lubov99d@mail.ru (L.S.); lbalabanova@mail.ru (L.B.); shubina@piboc.dvo.ru (L.S.); makarieva@piboc.dvo.ru (T.M.); 2 Far Eastern Federal University, Russky Island, Vladivostok 690022, Russia; tekuteva.la@dvfu.ru (L.T.); oksana_son@bk.ru (O.S.)

**Keywords:** sponge *Monanchora pulchra*, pentacyclic guanidine alkaloids, GH36 α-galactosidase, GH109 α-*N*-acetylgalactosaminidase, slow-binding irreversible inhibitor, monanchomycalin B, monanhocidin A, normonanhocidin A

## Abstract

The effect of monanchomycalin B, monanhocicidin A, and normonanhocidin A isolated from the Northwest Pacific sample of the sponge *Monanchora pulchra* was investigated on the activity of α-galactosidase from the marine γ-proteobacterium *Pseudoalteromonas* sp. KMM 701 (α-PsGal), and α-*N*-acetylgalactosaminidase from the marine bacterium *Arenibacter latericius* KMM 426^T^ (α-NaGa). All compounds are slow-binding irreversible inhibitors of α-PsGal, but have no effect on α-NaGa. A competitive inhibitor d-galactose protects α-PsGal against the inactivation. The inactivation rate (*k*_inact_) and equilibrium inhibition (*K*_i_) constants of monanchomycalin B, monanchocidin A, and normonanchocidin A were 0.166 ± 0.029 min^−1^ and 7.70 ± 0.62 μM, 0.08 ± 0.003 min^−1^ and 15.08 ± 1.60 μM, 0.026 ± 0.000 min^−1^, and 4.15 ± 0.01 μM, respectively. The 2D-diagrams of α-PsGal complexes with the guanidine alkaloids were constructed with “vessel” and “anchor” parts of the compounds. Two alkaloid binding sites on the molecule of α-PsGal are shown. Carboxyl groups of the catalytic residues Asp451 and Asp516 of the α-PsGal active site interact with amino groups of “anchor” parts of the guanidine alkaloid molecules.

## 1. Introduction

*O*-glycoside hydrolases are involved in the degradation of various poly- and oligosaccharides that serve as a source of carbon and energy for organism’s growth, as well as performing various functions in organisms. Modification or blocking of these functions by powerful selective inhibitors underlies the treatment of a number of infectious diseases, malignant tumors and genetic disorders [1]. Inhibitors of enzymes are molecules that reduce or completely block the catalytic activity of an enzyme, causing either complete death of a cell or modification in the metabolic pathways. The marine sponges are important sources of enzyme inhibitors [2,3].

α-d-galactosidases (α-d-galactoside galactohydrolases, EC 3.2.1.22) catalyze the hydrolysis of non-reducing terminal α-d-galactose (Gal) from α-d-galactosides, galactooligosaccharides and polysaccharides. α-d-galactosidases are widespread among terrestrial plants, animals, human organs and tissues, as well as microorganisms [4]. The enzymes occur frequently in marine bacteria, especially in γ-Proteobacteria and Bacteroidetes [5,6,7,8]. The marine enzyme α-d-galactosidase (α-PsGal) was isolated from the cold-adaptable marine bacterium *Pseudoalteromonas* sp. KMM 701 inhabiting in the cold water of the Sea of Okhotsk [9]. The enzyme attracted our attention due to its ability to reduce the serological activity of B red blood cells [9]. The enzyme also interrupted the adhesion of *Corynebacterium diphtheria* to buccal epithelium cells at the neutral pH [10], and regulated the growth of biofilms of some pathogenic bacteria [11]. According to the carbohydrate active enzymes’ classification (CAZy) [12] that is based on the amino acid sequence, α-PsGal belongs to the glycoside hydrolases (GHs) family 36.

α-*N*-Acetylgalactosaminidases (EC 3.2.1.49) catalyze the hydrolysis of the terminal α-linked *N*-acetylgalactosamine residues from the non-reducing ends of various complex carbohydrates and glycoconjugates. In the marine environment, α-*N*-acetylgalactosaminidases have been isolated from the liver and digestive organs of marine invertebrates, fishes, and marine bacteria of the genus *Arenibacter* [13,14]. The α-*N*-acetylgalactosaminidase from the marine bacterium *Arenibacter latericius* KMM 426^T^ (α-NaGa) was successfully applied for the complete conversion of A- into *O*-erythrocytes [15]. According to the CAZy classification, α-NaGa belongs to the GH109 family and is NAD^+^-dependent *O*-glycoside hydrolase as α-*N*-acetylgalactosaminidase from a clinical pathogen *Elizabethkingia meningoseptica* [14]. Thus, α-PsGal and α-NaGa were found to be potential tools for blood transfusion as well as for structural studies in glycobiology and infection diseases. Therefore, screening and studying the natural inhibitors of these enzymes should be helpful for understanding the molecular machinery of their function and designing a method for their removal from the reaction for medical purposes.

Some of the secondary metabolites from marine sponges, which are biologically active compounds, were found to be applicable for pharmacology as the inhibitors of different classes for enzymes [3,16]. To date, a number of alkaloids of unique structures have been isolated from the marine sponge *Monanchora* sp. [17]. Their antitumor activity and mechanism of action have been shown [18,19,20,21,22]. The effect of pentacyclic guanidine alkaloids monanchomycalin B, monanchocidin A and normonanchocidin A isolated from the marine sponge *Monanchora pulchra* on the activity of exo-β-1,3-d-glucanases from the marine filamentous fungus *Chaetomium indicum* and endo-β-1,3-d-glucanase LIV from the marine bivalve mollusk *Spisula sachalinensis* was investigated [23]. In the present study, we focus our attention on the effect of the marine sponge secondary metabolites with a good therapeutic potential on the activity of two well-characterized α-glycosidases to elucidate the mechanism of their inhibitor action.

The present article aimed to compare of the effects of monanchomycalin B, monanchocidin A and normonanchocidin A on the activities of recombinant α-galactosidase from the marine bacterium *Pseudoalteromonas* sp. KMM 701 of the GH36 family and α-*N*-acetylgalactosaminidase from the marine bacterium *Arenibacter latericius* KMM 426^T^ of the GH109 family.

## 2. Results and Discussion

### 2.1. Identification of the Compounds

**Figure 1:**
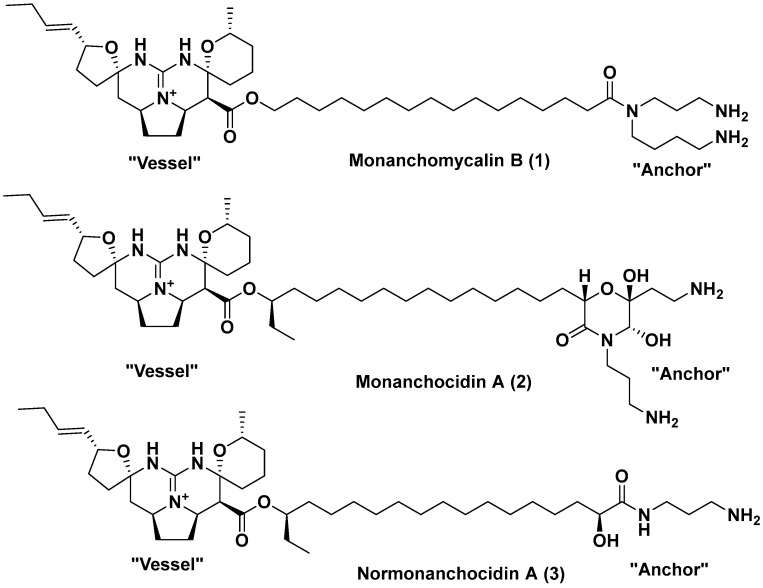
Structural formulas of pentacyclic guanidine alkaloids. “Vessel” part is on the left, and the “anchor” part is on the right of the molecule formula.

The samples of the marine sponge *M. pulchra* were collected in the Sea of Okhotsk (Kuril Islands region). The ethanol (EtOH) extract of the sponge *M. pulchra* sample N 047-243 was concentrated. The ethanol-soluble materials were further subjected to flash column chromatography on YMC*GEL ODS-A and high-performance liquid chromatography (HPLC) to obtain the pure monanchomycalin B (1). Monanhocidin A (**2**) and normonanhocidin A (**3**) were isolated from the EtOH extract of the sponge *M. pulchra* sample N 047-28 by the same method. The structure of the compounds **1**, **2** and **3** were assigned through the comparison of their spectral data with those reported in the references [20,21,22], respectively. Structural formulas of the pentacyclic guanidine alkaloids are shown on Figure 1.

The compounds isolated from the sponge *M. pulchra* have the same “vessel” part and differ in the structure of the “anchor” part of the molecule. The “anchor” part is presented by spermidine residue in monachomycalin B (**1**), by the tetra-substituted morpholinone derivative in monanchocidin A (**2**), and by the monosubstituted diaminopropane in normonanchocidin A (**3**).

### 2.2. Effect of Monanchomycalin B, Monanchocidin A, and Normonanchocidin A on Activity of Two Glycosidases

The results of the pretreatment of two marine bacterial glycosidases with pentacyclic guanidine alkaloids within 30 min (Table 1) showed that all three compounds inhibited the activity of recombinant GH36 α-PsGal and had no effect on the recombinant GH109 α-NaGa.

It was previously shown that all three compounds significantly activated *Sps*LamIV endo-β(1→3)-d-glucanase of the mollusk *Spisula sachalinensis* and completely inhibited *Chin*Lam exo-β(1→3)-d-glucanase of the marine fungus *Chaetomium indicum* [23].

We have shown with the example of monanchomycalin B that pentacyclic guanidine alkaloids irreversibly inactivate the α-PsGal. The activity of the enzyme did not recover after dialysis against the buffer solution for 72 h (Table 2). The decrease of free enzyme activity by 2.6 times was observed, probably, due to the enzyme α-PsGal thermolability [24] and instability at the low concentrations (data not shown).

**Figure 2:**
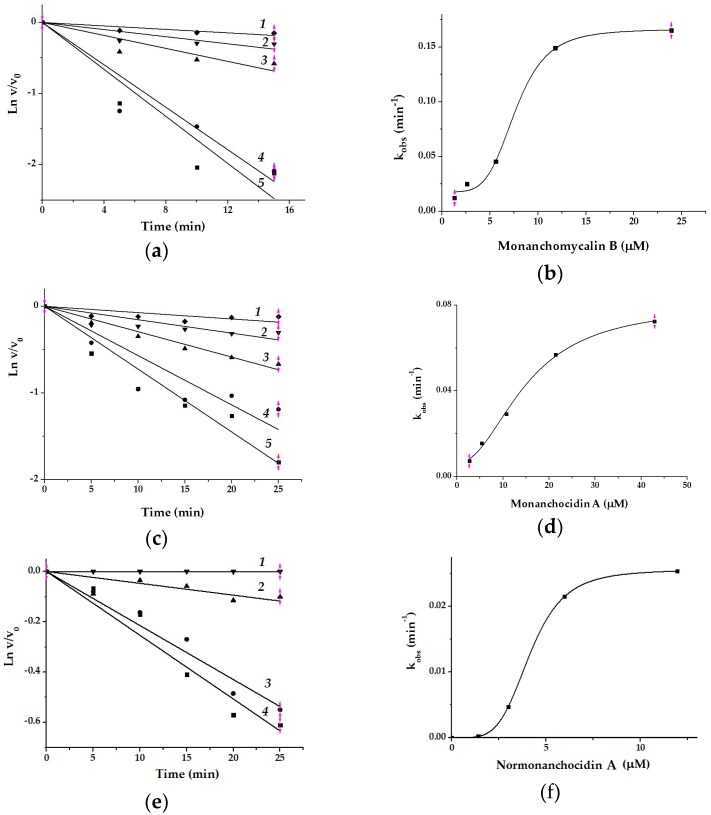
The results of kinetic studies of the α-PsGal inactivation by pentacyclic guanidine alkaloids: (**a**) the kinetic change of the residual activity of the enzyme (v/v_0_) in semilogarithmic coordinates at 1.3 μM (1), 2.66 μM (2), (3) 5.69 μM, (4) 11.8 μM, and (5) 23.9 μM of monanchomycalin B; (**b**) the inactivation rate constants (*k*_obs_) dependence on the concentrations of monanchomycalin B; (**c**) the kinetic change of the residual activity of the enzyme in semilogarithmic coordinates at 2.7 μM (1), 5.4 μM (2), (3) 10.7 μM, (4) 21.4 μM, and (5) 42.9 μM of monanchocidin A; (**d**) the inactivation rates (*k*_obs_) dependence on the concentrations of monanchocidin A; (**e**) the kinetic change of the residual activity of the enzyme in semilogarithmic coordinates at 1.49 μM (1), 2.98 μM (2), 5.97 μM (3), and 11.9 μM (4) of normonanchocidin A; (**f**) the inactivation rates (*k*_obs_) dependence on the concentrations of normonanchocidin A. All of the experiments were performed in duplicates.

The study of the inhibitory effect of monanchomycalin B, monanchocidin A, and normonanhocidin A at different concentrations and incubation times showed that the IC_50_ values of compounds decreased with increasing of the incubation time of α-PsGal with inhibitors (data not shown). The results of kinetic studies on the α-PsGal inactivation by pentacyclic guanidine alkaloids are shown on Figure 2. The curves of the dependences of the residual activity v/v_0_ on the time in semilogarithmic coordinates are shown in Figure 2a,c,e.

The α-PsGal inactivation developed relatively slowly, within a few minutes under these experimental conditions. In this case, the inhibitory activity of the compounds can be more accurately described by the inactivation rate constant (*k*_inact_, min^–1^) and equilibrium inhibition constant *K*_i_ [25]. The values of *k*_obs_ increased together with the compound concentrations. Sigmoid curves of *k*_obs_ dependences on concentration of the inhibitors (Figure 2b,d,f) mean that the process of the enzyme (E) inactivation by slowly-binding irreversible inhibitors (I) has a cooperative character, and occurs in two stages: (i) the formation of a reversible enzyme-inhibitor complex [E I^n^] and (ii) irreversible inactivation of the enzyme in the E‒I^n^ complex. The kinetic Equation (1) describes the irreversible slow inhibition of α-PsGal under the action of the pentacyclic guanidine alkaloids:

*K*_i_  *k*_inact_ E + nI ⇌ [E I^n^] → E‒I^n^, (1)

where n is coefficient of cooperativity, which is interpreted as the number of identical binding sites; *K*_i_ is an equilibrium constant of inhibition (μМ). The experimental dependences of *k*_obs_ on the concentration of the compounds (I) (Figure 2b,d,g) are approximated by the Hill’s Equation (2).

*k*_obs_ = *k*_inact_ I^n^/(*K*_i_^n^ + I^n^), (2)

The results of the experimental data fitting with theoretical curves are shown in Table 3.

According to the values of *K*_i_ and *k*_inact_, the alkaloids can be arranged in descending of binding-affinity as normonanchocidin A > monanchomycalin B > monanchocidin A, but in increasing inactivation rate in the following order: monanchomycalin B > monanchocidin A> normonanchocidin A.

**Figure 3:**
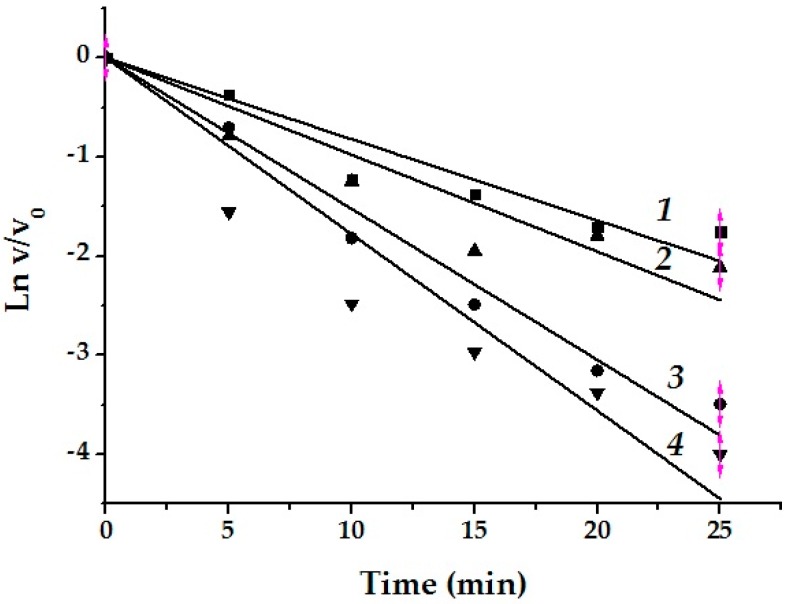
Protection of α-PsGal activity by d-galactose (0.7 mM) against monanchomycalin B inactivation: curves 1 and 2 show the effect of the enzyme activation rate on incubation time with the inhibitor (11.4 μM and 14.2 μM, respectively) in the presence of d-galactose in semi-log coordinates; curves 3 and 4 represent the rates of enzyme inactivation at the same inhibitor concentration and incubation time without d-galactose. All of the experiments were performed in duplicates.

Thus, based on the results of the kinetic studies, we have suggested that the pentacyclic guanidine alkaloids are slow-binding inhibitors for α-PsGal similarly to the *Chin*Lam glucanase [23]. It is accepted that slow-binding inhibition is observed whenever an enzyme-inhibitor complex forms or undergoes further conversion, at a slower rate relative to the overall reaction rate [26]. Inhibitors of peptidases [27], monoamine oxidases, and acetylcholinesterases [28,29] are examples of the slow-binding. Previously, the property of monanchocidin A as a slow-acting biologically active compound was shown for cancer cells [30]. For α-PsGal, the chlorine and bromine echinochrome derivatives from a sea urchin have been previously shown to be slow-binding inactivators as well [31]. Moreover, we have found that the inhibition rate increases with the binding of at least four molecules of the compounds.

d-galactose being a competitive inhibitor for α-galactosidases of GH36 family [32,33] decreased the activity of α-PsGal on 50% at 0.7 mM. The active-site-directed nature of the inactivation was proven by demonstration of the enzyme’s protection against inactivation by d-galactose (Figure 3).

From the Figure 3, it is evident that this monosaccharide significantly protects α-PsGal from the inactivation. 

Regardless of the inhibitor concentration, *k*_obs_^Gal^ decreased on average by 50% in the presence of the reaction product d-galactose (Table 4). The monosaccharide partially protects the enzyme from inactivation. This suggests that the inhibitor interacts with the enzyme molecule in the region of the active center.

Taking into account that the active center of the enzyme and the “vessel” part of the molecules of the compounds are identical in all the experiments, their inhibitory properties towards α-PsGal are determined by the structure of the “anchor” part. In this case, the diaminopropane residue has the greatest affinity, but more slowly penetrates to the active center of the GH36 α-PsGal from the marine bacterium. The monosubstituted diaminopropane has been shown to be also the best inhibitor for the *Chin*Lam exo-(1→3)β-d-glucanase from a marine fungus as well [23]. However, these compounds did not show inhibitor properties towards the GH109 α-NaGa from the marine bacterium *A. latericius* as well as the GH16 endo-(1→3)β-d-glucanase from the marine bivalve mollusk *S. sachalinensis* [23].

### 2.3. Theoretical Model of the Guanidine Alkaloids Complexes with α-Galactosidase

The enzyme α-PsGal is a typical *O*-glycoside hydrolase of the GH36 family. It was previously shown that its molecule consists of two identical subunits [9,10]. One subunit is a three-domain protein. The active center is located in the central (β/α)_8_ domain. Asp 451 and Asp 516 are catalytic residues [24].

**Figure 4:**
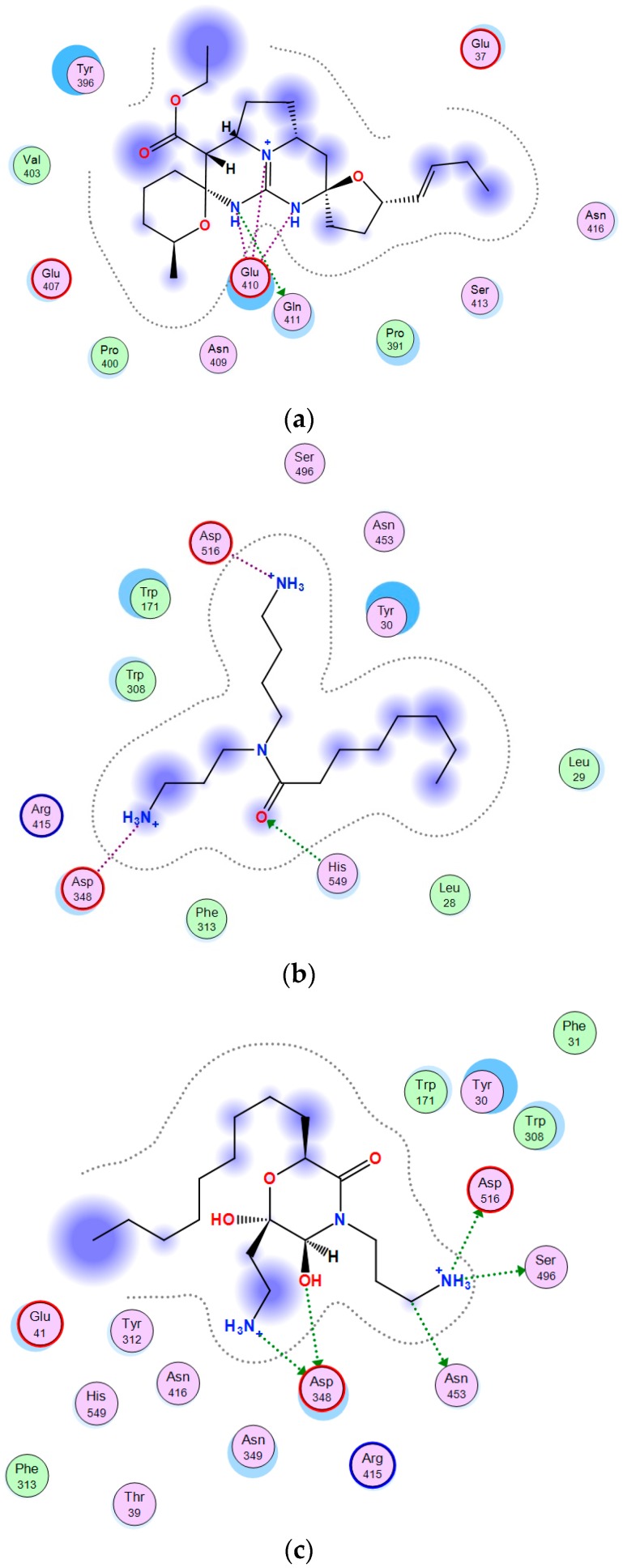

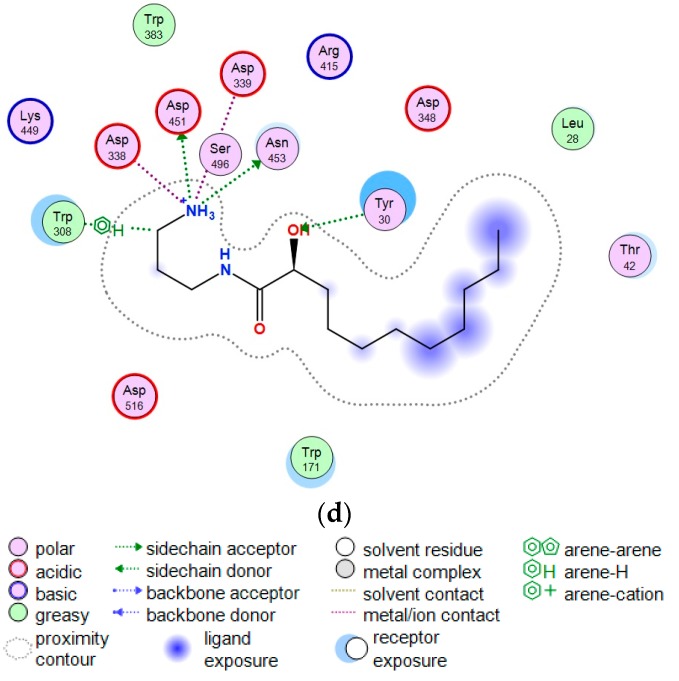
2D-diagrams of the α-PsGal complexes with the guanidine alkaloids: (**a**) 2D-diagram of α-PsGal—“vessel” part complex; (**b**) 2D-diagram of the α-PsGal-spermidine residue of monachomycalin B; (**c**) 2D-diagram of the α-PsGal-tetra-substituted morpholinone derivative of monanchocidine A; (**d**) 2D-diagram of α-PsGal—the monosubstituted diaminopropane of normonanchocidine A.

Figure 4 shows 2D-diagrams of the α-PsGal complexes with the guanidine compounds. The “vessel” part identical for the all compounds (Figure 4a), and the “anchor” parts of monanchomycalin B (Figure 4b), monanchocidin A (Figure 4c), and normonanchocidin A (Figure 4d) complexes with the active center of α-PsGal were built by molecular docking of the program Molecular Operating Environment version 2018.01 (MOE) [34].

The “anchor” parts of the compounds are the spermidine residue in monachomycalin B, tetra-substituted morpholinone derivative in monanchocidine A, and monosubstituted diaminopropane in normonanchocidine A.

Two different binding sites for the “vessel” and “anchor” parts of alkaloids in the molecule of α-PsGal were found. The carboxyl groups of the catalytic residues Asp451 and Asp516 in the active site of α-PsGal take part directly in the interaction with amino groups of “anchor” parts of the compounds.

The molecules of the test compounds consist of two polar nitrogen-containing residues connected by hydrophobic polymethylene chains. In this case, the “anchor” part of the molecule is very mobile. Based on the simulation results, the “vessel” part of the molecule binds near the crater of the active center and does not influence the activity of the enzyme, but directs and promotes an increase in the affinity of the “anchor” part; thus, the binding of the latter occurs more slowly and leads to the loss of enzyme activity. d-galactose located in the active center prevents the spermidine residue of monachomycalin B from entering to the catalytic site what can slow down the inactivation of the enzyme (Figure S1). In accordance with the results of a 3D-superposition of the α-PsGal active site with d-galactose and anchor parts of the compounds, the monosubstituted diaminopropane of normonanchocidine A penetrates most deeply into the pocket of the active center of α-PsGal (Figure S1c).

## 3. Materials and Methods

### 3.1. Materials

The 4-nitrophenyl-α-d-galactopyranoside (pNP-α-Gal), d-galactose (Gal), and 4-nitrophenyl-α-*N*-acetylgalactosaminide (pNP-α-NAcGal) were purchased from Sigma-Aldrich Chemical Company (St. Louis, MO, USA). Encyclo DNA-polymerase and enterokinase were purchased from Evrogen JSC (16/10 Miklukho-Maklaya str., Moscow, Russian Federation). Nco I and Sal I were purchased from New England Biolabs (NEB), Ipswich, MA, USA. pET 40 b(+) plasmid was purchased from Invitrogen, Carlsbad, CA, USA. Bacto-tryptone, sorbitol, MgCl_2_, KH_2_PO_4_, kanamycin, glycerin phenyl methanesulfonyl fluoride PMSF were purchased from (Helicon, Moscow, Russian Federation, Kutuzovsky Prospect, 88). Sodium phosphates one- and two-substituted were purchased from PanReac AppliChem GmbH (Ottoweg 4, Darmstadt, Germany). IMAC Ni^2+^ Sepharose, Q-Sepharose, Mono-Q, and Superdex-200 PG were purchased from GE Healthcare (Uppsala, Sweden). EtOH, trifluoroacetic acid (TFA), CD_3_OD were from the Russian Federation.

### 3.2. Experimental Equipment

Optical rotation was measured on Perkin-Elmer 343 polarimeter (Waltham, MA, USA). The ^1^H and ^13^C nuclear magnetic resonance (NMR) spectra were recorded on Avance III-700 spectrometer (Bruker BioSpin GmbH, Silberstreifen 4, D-76287 Rheinstetten/KarlsruheIpswich, Germany) at 700 and 175 MHz, respectively. The chemical shifts were correlated in accordance with the CD_3_OD signals (δ_H_ 3.30/δ_C_ 4 9.60). Electrospray ionization (ESI) mass spectra (including HRESIMS) were measured using a Bruker Impact II Q-TOF mass spectrometer (Bruker Daltonics, Bremen, Germany). HPLC was performed using Shimadzu instrument (Shimadzu Corporation, Kyoto, Japan) with a diffraction refractometer RID-DE14901810 and YMC-ODS-A column (YMC CO., LTD., Kyoto, Japan). Microplate spectrophotometer (BioTek Instruments, Highland Park, Winooski, VT, USA) was used for measuring of optical density at 400 nm (D_400_).

### 3.3. Collection and Identification of Sponge Material 

Samples of the sponge *Monanchora pulchra* N 047-028 and N 047-243 were collected by the dredging method during the 47th scientific cruise of the R/V *Akademik Oparin* in August 2015 at the Chirpoi (49°24.1 N, 154°17.8 E, depth of 139 m) and Onekotan, the Kuril Islands (49°24.1 N and 154°17.8 E, depth of 135 m). Identification of sponges was performed by V.B. Krasokhin. The voucher specimens are kept in the collection of G.B. Elyakov Pacific Institute of Bioorganic Chemistry, Far Eastern Branch, Russian Academy of Sciences (www.piboc.dvo.ru).

### 3.4. Isolation and Purification of Compounds

The freshly collected *M. pulchra* samples (N 047-243 and N 047-28) were extracted with EtOH and a part of which (30 mL) was concentrated in vacuo. The residual part was chromatographed on a microcolumn (10 × 12 mm) with YMC*GEL ODS-A reversed-phase sorbent (75 µm) using aqueous EtOH (40%), and then EtOH (65%)–H_2_O (35%)–TFA (0.1%). The eluates with TFA were evaporated. The compounds were isolated by HPLC using YMC-ODS-A column (250 × 10 mm) and EtOH (65%)–H_2_O (35%)–TFA (0.1%) to afford pure compound **1** (4.0 mg) from the sample 047-243, as well as compounds **2** (0.8 mg), and **3** (0.8 mg) from the sample 047-28.

Monanchomycalin B (**1**): high-resolution electrospray ionisation mass spectrometry (HRESI MS) *m/z* 785.6259 [M + H]^+^, (calcd. for C_45_H_81_N_6_O_5_: 785.6263); 

Monanchocidin A (**2**): HRESI MS *m/z* 859.6267, [M + H]^+^, (calcd. for C_47_H_83_N_6_O_8_: 859.6267);

Normonanchocidin A (**3**): HRESI MS *m*/*z* 758.5792, [M + H]^+^, (calcd. for C_43_H_76_N_5_O_6_: 758.5790).

### 3.5. Production of Recombinant Enzymes

#### 3.5.1. Production and Purification of Recombinant α-d-galactosidase

The recombinant wild-type α-d-galactosidase α-PsGal was produced as described earlier [35]. The plasmid DNA pET-40b(+) containing insertion of the gene from the marine bacterium *Pseudoslteromonas* sp. KMM 701 encoding α-PsGal was transformed in the *Escherichia coli* strain Rosetta (DE3). Heterological expression was carried out at optimal conditions as described previously [36]. Purification of the recombinant α-PaGal was performed according to the procedures described in the reference [35].

#### 3.5.2. Production and Purification of Recombinant α-Nacetylgalactosaminidase

The recombinant wild-type α-Nacetylgalactosaminidase α-NaGa was produced as described earlier [35]. The plasmid DNA pET-40b(+) containing insertion of the gene from the marine bacterium *Arenibacter latericius* KMM 426^T^ encoding α-NaGa was transformed in the *Escherichia coli* strain Rosetta (DE3). Heterological expression was carried out at optimal conditions as described previously [37].

The new purification procedure was modified and carried out at 4 °C. The cleared supernatant containing α-NaGa and 20% glycerol was loaded directly onto a Ni-sepharose column (5 cm × 36 cm) equilibrated with the buffer A (10 mM NaH_2_PO_4_, 10 mM Na_2_HPO_4_, 0.5 M NaCl, 5 mM imidazole, 20% glycerol, pH 8.0). The recombinant protein was eluted with the 5–500-mM linear imidazole gradient. The eluted fractions were analyzed, collected and dialyzed against the buffer B (10 mM NaH_2_PO_4_, 10 mM Na_2_HPO_4_, 50% glycerol, pH 8.0). Then, the protein solution was loaded onto a column (2 cm × 15 cm) with an ion-exchange resin Source 15Q equilibrated with the buffer B. The recombinant protein was eluted with the 0–1.5 M linear NaCl gradient. The fractions exhibiting the activity of α-NaGa were collected and examined using sodium dodecyl sulphate polyacrylamide gel electrophoresis (SDS-PAGE).

#### 3.5.3. Enzyme and Protein Assays

The activity of α-PsGal and α-NaGa were determined by increasing the amount of p-nithrophenol (pNP). The mixtures containing 50 μL of an enzyme solution and 100 μL of a substrate solution (1 mg/mL) in 0.05 M sodium phosphate buffer (pH 7.0) were incubated at 20 °C during 5 min for α-PsGal and 30 min for α-NaGa. The reactions were stopped by the addition of 150 μL of 1 M Na_2_CO_3_. One unit of the activity (U) was determined as the amount of an enzyme that releases 1 μmol of pNP per 1 min at 20 °С. The amount of the released pNP was determined spectrophotometrically (ε_400_ = 18,300 M^−1^ cm^−1^). The specific activity was calculated as U/mg of protein. The protein concentration was determined by the Bradford method calibrated with BSA as a standard [38]. Buffer solutions of α-PsGal (0.1 U/mL) and α-NaGa (0.05 U/mL) were used in the further experiments.

### 3.6. Effects of Pentacyclic Guanidine Alkaloids on Glycosidases of Marine Bacteria

#### 3.6.1. The Effects of Monanchomycalin B, Monanchocidin A, and Normonanchocidin A on Glyctosidases

To study the effect of pentacyclic guanidine alkaloids on α-PsGal and α-NaGa, 25 μL of an aqueous solution of monanchomycalin B, monanchocidin A, or normonanchocidin A (1 mg/mL) was mixed with 50 μL of the enzyme solutions in wells of the 96-cell plates and incubated for 30 min. Reactions were initiated by the addition of 75 μL of substrate solutions (p-nithrophenil galactopyranoside (pNP-α-Gal) for α-PsGal and p-nithrophenil N-acetylgalactosaminide (pNP-α-NAcGal) for α-NaGa) in 0.05 M sodium phosphate buffer (pH 7.0). The reaction mixture was incubated at 20 °C for 2–30 min in the final volume 150 μL, and then 150 μL of 1M Na_2_CO_3_ solution was added to the incubation mixture to stop the reaction. Each reaction mixture was prepared in duplicate. The absorbance was measured at 400 nm. Results were read with a Gen5 and treated with ExCel software. The activity of α-PsGal or α-NaGa was determined as described above. The residual activity was calculated as the ratio v/v_0_ (%), where v is the enzyme activity in the presence of an inhibitor, and v_0_ is the enzyme activity in the absence of an inhibitor. The v_0_ was taken for 100%.

#### 3.6.2. The Irreversibility of Monanchomycalin B Inhibition

To determine the reversibility of the inhibition of the α-PsGal activity, 40 μL (18 μM, H_2_O) of the monanchomycalin B solution was added to 60 μL of the enzyme solution; the mixture was incubated for 60 min. Two volumes of 20 μL were taken from the reaction mixture, 380 μL of a pNP-α-Gal solution (3.32 mM, in probe was ~5 K_m_) was added to each mixture, and then the reaction was stopped by addition of 0.6 mL of 1M Na_2_CO_3_ after 30 min of incubation. The value of optical density at the wavelength 400 (OD_400_) was measured in the 1-cm cuvette. The activity of α-PsGal was determined as described above. The remaining 60 μL of the reaction mixture was dialyzed against 1 L of 0.02 M sodium phosphate buffer (pH 7.0) for 72 h at 4 °C. To estimate the dilution, the volume of the reaction mixture after dialysis was measured. The enzyme activity was determined as described above and recalculated taking the dilution into account (1.7 times). A sample of α-PsGal untreated by monanchomycalin B (60 μL of the enzyme solution and 40 μL of H_2_O) was used as a control. The experiment was carried out in two replicates. The residual activity was calculated as described above. 

#### 3.6.3. The Assay of α-PsGal Inhibition by Monanchomycalin B, Monanchocidin A, and Normonanchocidin A

For the α-PsGal inhibition assay, 50 μL of the enzyme solution in 0.05 M sodium phosphate buffer (pH 7.0) were placed in the cells of the 96-well plate with 10 μL of a compound water solution at various concentrations in probes (186, 92.8, 46.4, 23.2, 11.6, 5.8, 2.9, 1.3, 0 μM were for monanchomycalin B; 171.5, 85.7, 42.9, 21.4, 10.7, 5.4, 2.7, 0 μM were for monanchocidin A, and 191.1, 95.6, 47.8, 23.9, 11.9, 6.0, 3.0, 1.5, 0 μM were for normonanchocidin A), and incubated for each concentration during 5, 10, 15, 20, and 25 min. The enzyme reaction was initiated by the addition of 90 μL of the pNP-α-Gal solution (3.32 mM, ~5 K_m_ for probe) in 0.05 M sodium phosphate buffer (pH 7.0). The reaction mixtures were incubated for 2 - 15 min in the final volume 150 μL, then 150 μL of the water solution of Na_2_CO_3_ (1 M) were added to stop the reaction, and OD_400_ for the reaction mixtures were immediately measured by a microplate spectrophotometer. The time of each reaction was strictly monitored by stopwatch. The standard and residual activity v/v_0_ were calculated as described above.

#### 3.6.4. The Kinetic Parameters of Inactivation

The equilibrium inhibition constants (*K*_i_) and kinetic inactivation constants (*k*_inact_) were determined by the classical methods [39]. The inactivation of α-PsGal by the different concentrations of inhibitors (1.3–50 μM) was performed in 0.05 M sodium phosphate buffer (pH 7.0) at a temperature 20 °C. An aqueous solution (25 μL) of the compound at the different concentrations was added to 50 μL of the α-PsGal solution (0.2 U/mL), held for 5, 10, 15, 20, and 25 min at 20 °C, then 100 μL of the pNP-α-Gal solution was added and incubated for 2–15 min at 20 °C. The same conditions were used in the control reaction, but the inhibitor was replaced with distilled water. The reactions were stopped by the addition of 1 M Na_2_CO_3_ (150 μL); the amount of pNP formed in 1 min was determined as described above. The residual activity v/v_0_ was presented as a function of time. The pseudo-first-order rate constant of inactivation (*k*_obs_) was determined for each inactivator concentration as the slope of the v/v_0_ dependence on the incubation time in semilogarithmic coordinates. The ExCel software was used for these calculations. The second order rate constants for the inactivation process were determined by fitting the dependences of the *k*_obs_ values on the concentration of the inactivators to the Hill’s equations. An analysis of the curves and the choice of models for calculation of *K*_i_ (µM) and *k*_inact_ (min^–1^) were performed with the Origin 8.1 software (OriginLab, Northampton, MA, USA).

#### 3.6.5. Protection of α-PsGal Inactivation by d-galactose

The active-site-directed nature of the inactivation was confirmed by demonstrating protection against the inactivation by competitive inhibitor d-galactose. Inactivation mixtures (75 μL) containing 50 µL of the enzyme solution and 10 µL of d-galactose (0.7 mM in mixture) were preincubated for 15 min, then 15 µL of the monanchomycalin B solution (11.4 μM and 14.2 μM in mixture) were added and incubated at various time intervals as described above. The residual activity of the enzyme was assayed as described above.

### 3.7. Theoretical Models of α-PsGal Complexes with Guanidine Alkaloids

The target-template alignment customization of the modeling process and 3D model building of α-PsGalA (GenBank: ABF72189.2) were carried out using the Molecular Operating Environment version 2018.01 [37] package using the forcefield Amber12: EHT. The α-d-galactosidase from *Lactobacillus acidophilus* NCFM (Protein data bank (PDB) code: 2XN2) with a high-resolution crystal structure was used as a template. The evaluation of structural parameters, contact structure analysis, physical-chemical properties, molecular docking, and visualization of the results were carried out with the Ligand interaction and Dock modules in the MOE 2018.01 program. The results were obtained using the equipment of Shared Resource Center Far Eastern Computing Resource of Institute of Automation and Control Processes Far Eastern Branch of the Russian Academy of Sciences (IACP FEB RAS) [40].

## 4. Conclusions

For study the effect of the marine sponge metabolites with a therapeutic potential, we used two well-characterized α-glycosidases for justifying a possible mechanism of their inhibitor action. Monanchomycalin B, normonanchocidin A, monanchocidin A have been shown to be irreversible slow-binding inhibitors of the GH36 family α-galactosidase α-PsGal from the marine bacterium *Pseudoalteromonas* sp. КММ 701, but have no effect on the activity of the GH109 family α-NaGa from the marine bacterium *Arenibacter latericius* KMM 426^T^. The inhibitory ability of the alkaloids depends on the chemical structure of the anchor parts of their molecules. The alkaloids can be arranged in the descending order of the binding-affinity: normonanchocidin A > monanchomycalin B > monanchocidin A, and in the decreasing order of the inactivation rate: monanchomycalin B > monanchocidin A > normonanchocidin A. These highly active marine compounds selectively acted on the enzymes from the different structural GH families, binding to the electronegative areas of the protein surfaces formed mainly by carboxylic acid side groups in the active-site-directed manner. The well-characterized α-glycosidases of marine bacteria have been proved to be suitable models for characterizing the novel properties of the alkaloids.

## Figures and Tables

**Table 1:** Residual activity v/v_0_ (%) of the glycosidases after incubation with monanchomycalin B, monanchocidin A, or normonanchocidin A ^1^.

Enzyme	H_2_O	Monanchomycalin B	Monanchocidin A	Normonanchocidin A
α-PsGal	100	0.21	2.7	1.7
α-NaGa	100	101.5	98.5	83.0

^1^ Concentration of compounds in each sample was 0.2 mM, the enzyme was preincubated with an inhibitor for 30 min, 20 °C, pH 7.0.

**Table 2:** The activity of α-PsGal after treating with monanchomycalin B ^1^.

Monanchomycalin B (μM)	Residual Activity (%)	
	Before Dialysis	After Dialysis
0	100	38
18	0	0

^1^ The activity of α-PsGal after dialysis (72 h, 4 °C, 0.02 M sodium phosphate buffer (pH 7.0)) is presented considering the dilution of the enzyme sample. The results are average of three parallel measurements.

**Table 3:** The α-PsGal inhibition constants for monanchomycalin B, monanchocidin A, and normonanchocidin A.

Inhibitor	*k*_inact_ (min^−1^)	*K*_i_ (μM)	n
Monanchomycalin B	0.166 ± 0.029	7.70 ±0.62	4.64 ± 1.21
Monanchocidin A	0.08 ± 0.003	15.08 ± 1.60	2.1 ± 0.47
Normonanchocidin A	0.026 ± 0.000	4.15 ± 0.01	4.55 ± 0.02

**Table 4:** Protection of α-PsGal activity by d-galactose against monanchomycalin B inactivation.

Concentration (μM)	*k*_obs_ (min^−1^)	*k*_obs_^Gal^ (min^–1^) ^1^
11.4	0.152 ± 0.005	0.082 ± 0.006
14.2	0.178 ± 0.013	0.097 ± 0.008

^1^*k*_obs_^Gal^—in the presence of d-galactose.

## Supplementary Materials

The following are available online at http://www.mdpi.com/1660-3397/17/1/22/s1, Figure S1. 3D-superposition of the α-PsGal active site with d-galactose and spermidine residue of monachomycalin B (**a**), tetra-substituted morpholinone derivative of monanchocidine A (**b**) and monosubstituted diaminopropane of normonanchocidine A (**c**). Parts of guanidine alkaloids are shown as “ball and stick” with grey color, galactose shown as “stick” with yellow color. The molecular surface closed to the ligands is shown in pink (H-bonding), green (hydrophobic) and blue (mild polar).
