# Supplementary material for: Effect of Pentacyclic Guanidine Alkaloids from the Sponge Monanchora pulchra on Activity of α-Glycosidases from Marine Bacteria
Source: Mar Drugs. 2019 Jan 1;17(1):22. doi: 10.3390/md17010022 (PMC6356649; doi:10.3390/md17010022)
Supplement: Supplementary file 1 [file marinedrugs-17-00022-s001.pdf]

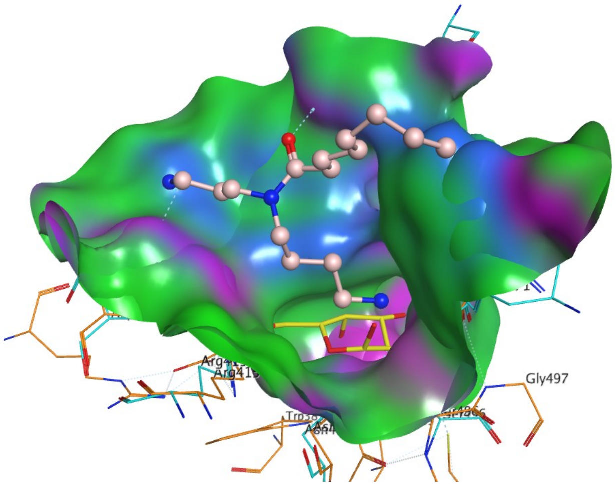
Gly497

(**a**)

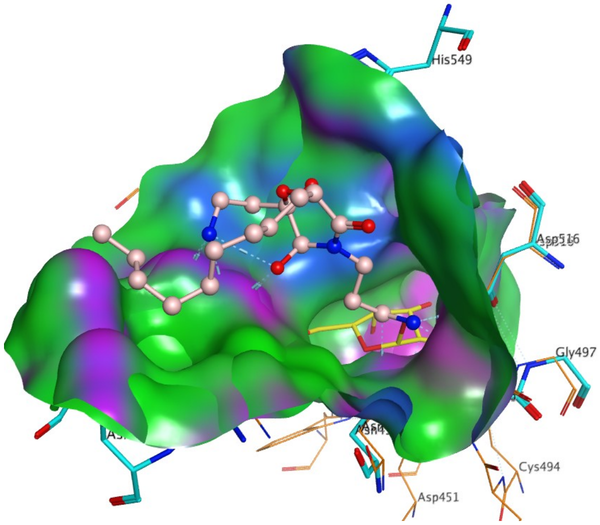
His549
Asp516
Gly497
Cys494
Asp451

(**b**)

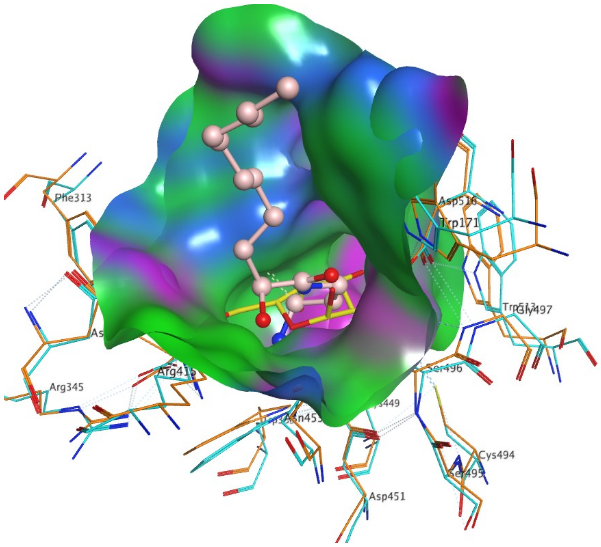
Phe313
Asp516
Trp171
Arg345
Ser496
Cys494
Asp451

(**c**)

Figure S1. 3D-superposition of the α-PsGal active site with galactose and spermidine residue of monachomycalin B (**a**), tetra-substituted morpholinone derivative of monanchocidine A (**b**) and monosubstituted diaminopropane of normonanchocidine A (**c**). Parts of guanidine alkaloids are shown as "ball and stick" with grey color, galactose shown as "stick" with yellow color. The molecular surface close to the ligands is shown in pink (H-bonding), green (hydrophobic) and blue (mild polar).
